# Proinflammatory Cytokine Modulates Intracellular Calcium Handling and Enhances Ventricular Arrhythmia Susceptibility

**DOI:** 10.3389/fcvm.2021.623510

**Published:** 2021-03-16

**Authors:** Yung-Nan Tsai, Ya-Wen Hsiao, Shien-Fong Lin, Yi-Hsin Chan, Yu-Cheng Hsieh, Wei-Hua Tang, An-Sheng Lee, Yu-Ting Huang, Hsing-Yuan Li, Tze-Fan Chao, Satoshi Higa, Tsu-Juey Wu, Shih-Lin Chang, Shih-Ann Chen

**Affiliations:** ^1^Institute of Clinical Medicine, National Yang-Ming University, Taipei, Taiwan; ^2^Division of Cardiology, Department of Medicine, Heart Rhythm Center, Taipei Veterans General Hospital, Taipei, Taiwan; ^3^Institute of Biomedical Engineering, National Chiao-Tung University, Hsin-Chu, Taiwan; ^4^Division of Cardiology, Chang Gung Memorial Hospital, Taoyuan, Taiwan; ^5^Department of Internal Medicine, Cardiovascular Center, Taichung Veterans General Hospital, Taichung, Taiwan; ^6^Division of Cardiology, Department of Internal Medicine, National Yang-Ming University Hospital, Yilan, Taiwan; ^7^Department of Medicine, Mackay Medical College, New Taipei, Taiwan; ^8^Division of Cardiology, Department of Pediatrics, Taipei Veterans General Hospital, Taipei, Taiwan; ^9^Faculty of Medicine, School of Medicine, National Yang-Ming University, Taipei, Taiwan; ^10^Cardiac Electrophysiology and Pacing Laboratory, Division of Cardiovascular Medicine, Makiminato Central Hospital, Urasoe, Japan

**Keywords:** alternans, the maximum calcium transient, IL-17 neutralizer, IL-17, ventricular arrhythmias

## Abstract

**Background:** The mechanism of Interleukin-17 (IL-17) induced ventricular arrhythmia (VA) remains unclear. This study aimed to investigate the effect of intracellular calcium (Ca_i_) handling and VA susceptibility by IL-17.

**Methods:** The electrophysiological properties of isolated perfused rabbit hearts under IL-17 (20 ng/ml, *N* = 6) and the IL-17 with neutralizer (0.4 μg/ml, *N* = 6) were evaluated using an optical mapping system. The action potential duration (APD) and Ca_i_ transient duration (Ca_i_TD) were examined, and semiquantitative reverse transcriptase-polymerase chain reaction analysis of ion channels was performed.

**Results:** There were longer APD_80_, Ca_i_TD_80_ and increased thresholds of APD and Ca_i_TD alternans, the maximum slope of APD restitution and induction of VA threshold in IL-17 group compared with those in IL-17 neutralizer and baseline groups. During ventricular fibrillation, the number of phase singularities and dominant frequency were both significantly greater in IL-17 group than in baseline group. The mRNA expressions of the Na^+^/Ca^2+^ exchanger, phospholamban, and ryanodine receptor Ca^2+^ release channel were upregulated, and the subunit of L-type Ca^2+^ current and sarcoplasmic reticulum Ca^2+^-ATPase 2a were significantly reduced in IL-17 group compared to baseline and IL-17 neutralizer group.

**Conclusions:** IL-17 enhanced Ca_i_TD and APD alternans through disturbances in calcium handling, which may increase VA susceptibility.

## Introduction

Ischemic ventricular arrhythmia (VA) is strongly associated with increased inflammatory activities (1). Although interleukin (IL)-17, the pro-inflammatory cytokine of the newly described T helper 17 (T_H_ 17) cell subset, has a major function in shielding the host anti extracellular pathogens, it promotes inflammation in autoimmune diseases and mediator of tissue inflammation (2, 3). Former studies have demonstrated that downregulated IL-17 expression inhibits the inflammatory response and improves heart function (4). On the other hand, an important role for IL-17 in post-myocarditis cardiac remodeling and the development to dilated cardiomyopathy was described (5). Our previous study showed that IL-17 treatment lead to fibrosis, collagen production, and apoptosis in the left ventricular (LV) tissue. Moreover, the study showed that increased IL-17 activates mitogen-activated protein kinase (MAPK) and thereby increases the expression of downstream target genes, including IL-6, tumor necrosis factor (TNF), C-C motif chemokine ligand 20 (CCL20), and C-X-C motif chemokine ligand 1 (CXCL1) (4). VA can be triggered through multiple electrophysiological mechanisms, including prolonged ventricular action potential duration (APD), slowed conduction, increased electrical restitution, and perturbed intracellular calcium (Ca_i_) signaling (6, 7). Therefore, inflammation and Ca_i_ handling are involved in VA. However, the function of IL-17 with regard to Ca_i_ alternans and VA susceptibility are yet unclear. Therefore, this study aimed to investigate the influence of IL-17 on Ca_i_ handling and VA susceptibility.

## Materials and Methods

### Surgical Preparation

The study protocol was reviewed and approved by the Institutional Animal Care and Use Committee of Taipei Veterans General Hospital. New Zealand white male rabbits (*N* = 30), weighing 2.5–3.5 kg, were used for optical mapping (*N* = 12) and extraction of RNA (*N* = 18). Rabbits were deeply anesthetized using intramuscular injection of a mixture of Zoletil 50 (10 mg/kg) and Xylazine (5 mg/kg). The subcutaneously at the incision site, we injected 2% Xylocaine (3 mL). An intravenous bolus of heparin (2,500 units) was administered to the rabbits to avoid intracardiac clot formation. The hearts of the experimental rabbits were exposed by median thoracotomy and pericardiotomy, and the rabbits were quickly sacrificed by manual excision of the beating hearts, which were directly submerged in cold with oxygenated Tyrode's solution of the following composition (mmol/L): Na^+^, 156.5; K^+^, 4.7; Ca^2+^, 1.5; H_2_${\text{PO}}_{4}^{-}$, 0.5; Cl^−^, 137; ${\text{HCO}}_{3}^{-}$, 28; glucose, 20 with a pH of 7.40 (4).

### Langendorff Preparation and Optical Mapping

The hearts were suspended on the cannula with silk tied through the ascending aorta. Deep insertion of the aorta into the perfusion cannula must be avoided because it can cause the compression of the coronary arteries. Continuous circulation and heart perfusion were preserved using a roller pump. The perfusion oxygenated Tyrode's solution flowed through the coronary arteries and returned through the coronary sinus was collected in a reservoir (thermostatically maintained at 37°C), from which the perfusion solution returned to the circulation system. The system was regulated to keep a constant perfusion pressure (30–60 mmHg under controlled conditions). Myocardial pseudo-electrocardiogram (pseudo-ECG) signals were monitored using 3 pseudo-ECG pins that were inserted into the ventricles. A mapping catheter was inserted and secured inside the right ventricular (RV) apex through pulmonary vein and RV. Using a camera optical mapping system, the epicardial activation patterns were studied during ventricular pacing (4, 6). The hearts were stained with RH237 (10 μmol/L, 0.4 μmol in 40 ml Tyrode's solution, from Invitrogen, Grand Island, NY) for membrane potential (Vm) mapping and with Rhod-2 AM (1.2 μmol/L, 0.18 μmol in Tyrode's solution, from Invitrogen, Grand Island, NY) for Ca_i_ mapping. Blebbistatin (15–20 μmol/L, from Tocris Bioscience, Minneapolis, MN) was used to inhibit cardiac contraction. We used cytochalasin-D (5 μmol/L), an excitation-contraction uncoupler to minimize motion artifacts. The hearts were excited using 2 light-emitting diode modules at a wavelength of 532 nm. The signals were recorded simultaneously using 2 MiCAM02 cameras (BrainVision, Tokyo, Japan). Optical signals were gathered at 2 ms/frame temporal resolution, acquired from 128 × 128 sites simultaneously over a 30 × 30 mm^2^ area in each aspect of those hearts. For each optical recording, data were acquired continuously for 2 s. Optical signals were processed with both spatial (3 × 3 pixels Gaussian filter) and temporal (3 frames moving average) filtering (7, 8).

### The Rationale for IL-17 and IL-17 Neutralizer Dosage

In our previous studies, IL-17 concentration (200 pg/ml) was measured from heart failure (HF) rabbit serum (4). To mimic the inflammatory process of HF, we used the dosage of IL-17 ranged from 20 ng/ml in Langendorff perfusion which is similar to the level of IL-17 in whole rabbit. Neutralizing antibody 0.4 **μ**g/ml was used for block of IL-17 receptor before IL-17 reperfusion (4). In addition, previous studies showed that IL-17 produced rapid phosphorylation of protein kinase B and ERK within 5 min, and it rapidly enhanced excitability (9). Therefore, IL-17 with dosage of 20 ng/ml would be adequate to study the influences of IL-17 on VA in Langendorff perfusion study, and dosage of 0.4 μg/ml in neutralizing antibody would be adequate to be an antidote. IL-17 neutralizer is a recombinant, high affinity, fully human IgG1/κ monoclonal antibody that selectively binds to and neutralizes IL-17. Binding of IL-17 by IL-17 neutralizer inhibits its interaction with the IL-17 receptor, thereby inhibiting the release of other proinflammatory cytokines, chemokines and mediators of tissue damage and reducing the contribution of IL-17 to these inflammatory diseases (10).

### Experimental Protocol and Electrophysiological Study

IL-17 (20 ng/mL, RPA063Rb01; Cloud-Clone Corp) was added and perfused for 10 min in the Langendorff-perfused normal rabbit heart (*N* = 6). For neutralizing experiments (*N* = 6), IL-17 neutralizing antibodies (0.4 *μ*g/mL, eBio64CAP17; eBioscience) were perfused for 10 min before the perfusion of IL-17 20 ng/mL for 10 min. There was no washout phase. The baseline group was normal rabbit heart before treatment. A bipolar electrode was inserted into the RV apex for pacing. This protocol comprised different pacing cycle lengths (PCL) ranging from 500 to 130 ms. Regarding each PCL, the S1 pacing train was obtained during steady-state S1 pacing (>50 beats after the onset of pacing), and then optical mapping data was recorded. APD_80_ and Ca_i_ transient duration (Ca_i_TD_80_) were measured at 80% repolarization which would avoid the undetermined baseline in phase 4 period (4, 6, 7). The F/F_0_ ratio was used to measure the relative concentration of Ca_i_, and the maximum Cai transient F/F_0_ were measured (6).

### APD and Ca_i_ Alternans During S1 Pacing

Rapid pacing protocol was performed, initially at cycle length of 500 ms, decremented by 50 ms every 8 beats until reaching a cycle length of 250 and 250 ms decremented by 10 ms until reaching a cycle length of 130 ms or the loss of 1:1 capture of the ventricles. The thresholds of APD and Ca_i_ alternans were defined by determining differences in local APD and Ca_i_ on consecutive beats (11).

### Induction of Ventricular Arrhythmia and Phase Mapping

We used Fast Fourier Transforms of pseudo-ECG (4 s in duration) to determine the dominant frequency (DF) of ventricular fibrillation (VF) at IL-17 and IL-17 neutralizer group. Phase mapping was performed to assess the location and development of phase singularities (PSs). PS observed on the phase maps was defined as a site with an ambiguous phase enclosed by pixels showing a continuous phase progression from –π to +π. Previous studies recommend that PSs are a robust alternative representation of wavebreaks (7), which serve as the source of VF. To quantify wavebreaks during VF, the numbers of PSs in the phase map were calculated manually every 10 frames for 1,000 frames in each episode of VF (7). VA inducibility was measured using eight-beat drive trains at 240-and 200-ms BCLs, followed by 1–3 ventricular extrastimuli. Single (S2), double (S2-S3), or triple (S2-S3-S4) premature stimuli were applied with a coupling interval of 160 ms (S2), 150 ms (S3), or 140 ms (S4), and gradually shortened in 5-ms steps until VA was induced or until the ventricular effective refractory period was reached. VA included VF, and tachycardia was defined as ≥4 consecutive ventricular ectopic beats at a cycle length ≤150 ms (ventricular tachycardia) or by unidentifiable and low-voltage QRS complexes (VF). The percentage of inducible VA episodes was counted as the ratio of induced VA episodes to the number of ventricular extra stimuli applied. This induction protocol was standardized across all experiments (4).

### Semiquantitative Reverse Transcription Polymerase Chain Reaction

Tissues were obtained from Langendorff perfusion normal group (*N* = 6), IL-17 neutralizer group (*N* = 6), and IL-17 group (*N* = 6). In IL-17 group, IL-17 20 ng/mL was perfused for 10 min. In IL-17 neutralizer group, IL-17 neutralizing antibodies 0.4 μg/mL was perfused for 10 min following the perfusion of IL-17 20 ng/mL for 10 min. The LV tissues using the RNeasy^®^ Fibrous Tissue Kit (Qiagen, Valencia, CA, USA), according to the manufacturer's protocol. Further, cDNA was synthesized using Prime Script^™^ Reverse Transcriptase (Takara Bio Inc., Kyoto, Japan) with a random hexamer from 5.0 μg of total RNA. The resulting cDNA was detected by polymerase chain reaction (PCR) via the DreamTaq Green PCR Master Mix (Thermo Scientific Inc., Waltham, MA, USA) for 40 cycles at an annealing temperature of 55°C with a Veriti^®^ 96-Well Fast Thermal Cycler (Applied Biosystems, Carlsbad, CA, USA). PCR products were visualized under UV light with ethidium bromide and quantified with Image-Pro Plus software. Primer sequences for PCR detection are provided in Supplementary Table 1.

### Data Analysis

APD was measured from the steepest deflection of the slope of phase 0 to the time of APD_80_. We used the SD of APD_80_ at all mapped pixels to measure the spatial heterogeneity of APD (7). Conduction velocity (CV) in squares (10 × 10 mm) located at the centers of the anterior aspects of LV was measured (4). The diastolic interval (DI) was measured from the APD_80_ of the prior beat to the current action potential onset. A restitution curve (RC) was plotted using APD_80_ against the preceding DI by S1 pacing (4, 6, 7). The maximum slope of RC was counted by first-order exponential fitting using ORIGIN software (Microcal) (12). The time constant of the Ca_i_ decay (tau value) was determined by a monoexponential least-squares fit. The detected longest S1 PCL threshold was spatially concordant alternans (SDA) threshold was defined (13). Positive coupling of Ca_i_-Vm alternans was defined as long APD corresponds to large Ca_i_ transient and negative coupling of Ca_i_-Vm alternans was defined as long APD corresponds to small Ca_i_ transient. The incidence of positive and negative coupling episodes was counted as the ratio of positive or negative coupling episodes of Ca_i_-Vm alternans to the number of pacing numbers applied (14).

### Statistical Analyses

Quantitative data were expressed as mean ± SD. Two-way repeated measures analysis of variance was used to compare the differences before and after IL-17 group in subgroups. A senior biostatistician performed the statistical analysis using SPSS version 17 (SPSS Institute Inc., Chicago, IL, USA). Furthermore, *p* < 0.05 was considered to be statistically significant.

## Results

### Effect of IL-17 on Ca_i_ Transient and APD/Ca_i_TD Prolongation

**Figure 1:**
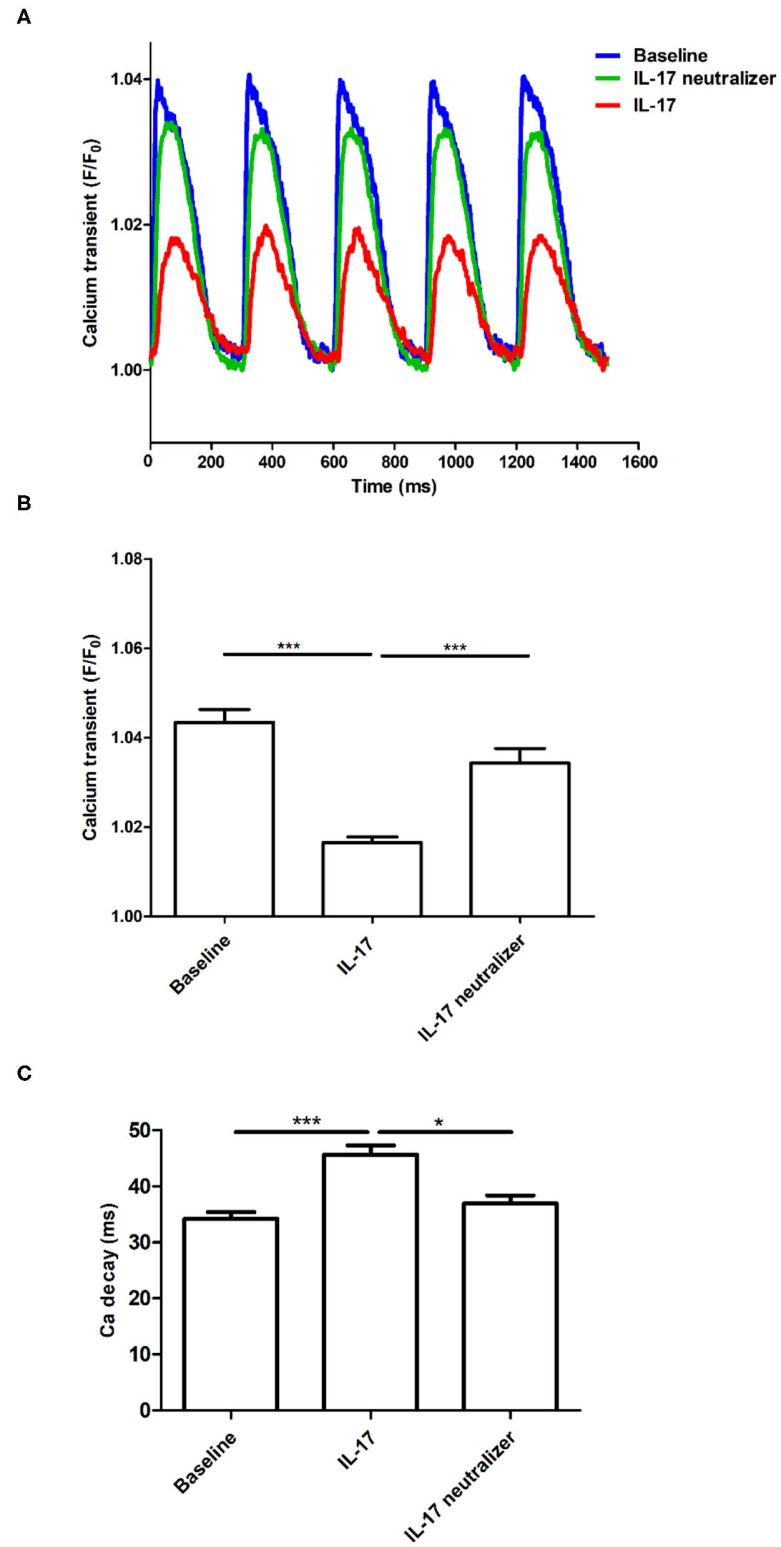
Effect of IL-17 infusion, IL-17 neutralizer infusion alone, and baseline on intracellular calcium transient. The maximum Ca^2+^ transient (F/F_0_) was decreased after IL-17 infusion compared with baseline **(A,B)**. Ca_i_ decay (tau value) was prolonged in the IL-17 group than that in the baseline and IL-17 neutralizer groups **(C)**. ^*^P < 0.05; ^***^P < 0.001. Ca_i_, intracellular calcium.

**Figure 2:**
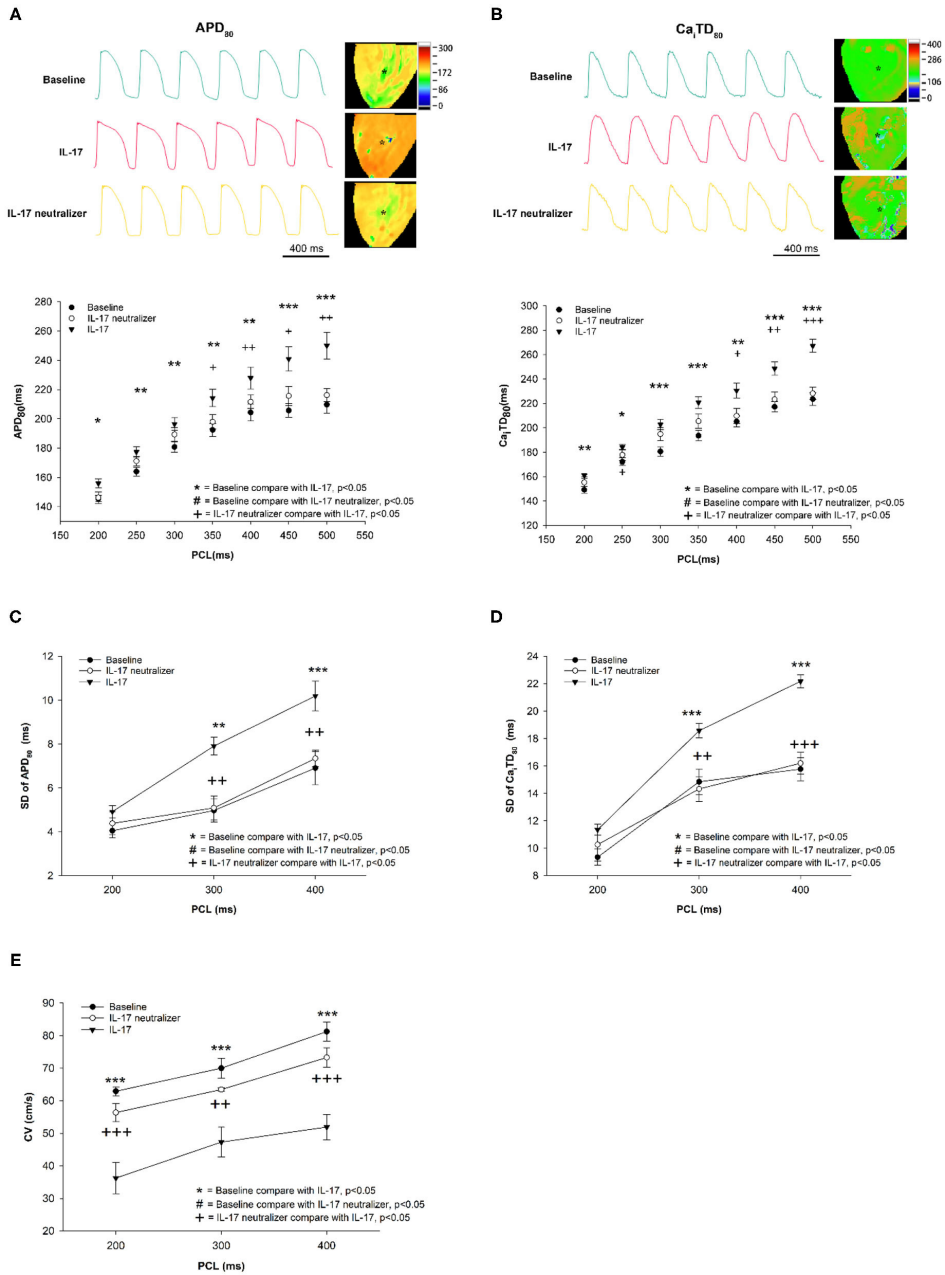
Effect of IL-17 on spatial heterogeneity of APD and Ca_i_TD in the baseline and IL-17 neutralizer groups. APD_80_ and Ca_i_TD_80_ **(A,B)**. SD of APD_80_ and SD of Ca_i_TD_80_ **(C,D)**. CV **(E)** in the LV during different PCLs. ^*^P < 0.05; ^**^P < 0.01; ^***^P < 0.001; ^+^*P* < 0.05; ^++^*P* < 0.01; ^+++^*P* < 0.001. APD, action potential duration; Ca_i_TD, calcium transient duration; APD_80_, action potential duration at repolarizations of 80%; Ca_i_TD_80_, calcium transient duration at repolarizations of 80%; CV, conduction velocity; PCL, pacing cycle length; LV, left ventricle.

Optical images were captured from the whole ventricle. The maximum Ca^2+^ F/F_0_ was recorded in LV during ventricular pacing at 300 ms PCL (Figure 1A). The maximum Ca^2+^ F/F_0_ was significantly reduced in the IL-17 group compared with the baseline and IL-17 neutralizer groups (1.01 ± 0.003, 1.04 ± 0.008, 1.03 ± 0.007, respectively; *P* < 0.001) (Figure 1B). No significant difference in maximum Ca^2+^ F/F_0_ was observed between the baseline and IL-17 neutralizer groups (*P* = 0.06). IL-17 had a lower Ca_i_ concentration accumulated over the time of the transient compared to baseline. The Ca decay time in the IL-17 group was prolonged than that in the baseline and IL-17 neutralizer groups (45.6 ± 1.6, 34.2 ± 1.2, and 37.0 ± 1.5 ms, respectively, *P* < 0.05) (Figure 1C). The effects of IL-17 on APD_80_ and Ca_i_TD_80_ at 300 ms PCL are shown in Figure 2. Compared with the baseline, APD_80_ and Ca_i_TD_80_ were prolonged in the IL-17 group (*P* < 0.05 at all PCL). APD_80_ was shorter in the IL-17 neutralizer group (*P* < 0.05 at PCL of 350–500 ms) than in the IL-17 group (Figure 2A). The IL-17 group had a longer Ca_i_TD_80_ than the baseline and IL-17 neutralizer groups at all PCL and PCL of 400–500 ms, respectively, (*P* < 0.05) (Figure 2B). No significant difference in APD_80_ and Ca_i_TD_80_ was be found between the baseline and IL-17 neutralizer groups (P = NS). Compared with the baseline, SD of APD_80_ and SD of Ca_i_TD_80_ were prolonged in the IL-17 group (*P* < 0.05 at all PCL). SD of APD_80_ was shorter in the IL-17 neutralizer group (*P* < 0.05 at PCL of 300–400 ms) than in the IL-17 group (Figure 2C). The IL-17 group had a longer SD of Ca_i_TD_80_ than the baseline and IL-17 neutralizer groups (*P* < 0.05 at PCL of 300–400 ms) (Figure 2D). No significant difference in SD of APD_80_ and Ca_i_TD_80_ was found between the baseline and IL-17 neutralizer groups (P = NS). IL-17 group had a decreased CV compared with baseline and IL-17 neutralizer group. Treatment of IL-17 neutralizer increased CV compared to that in IL-17 group (Figure 2E).

### Pacing-Induced APD and Ca_i_TD Alternans

**Figure 3:**
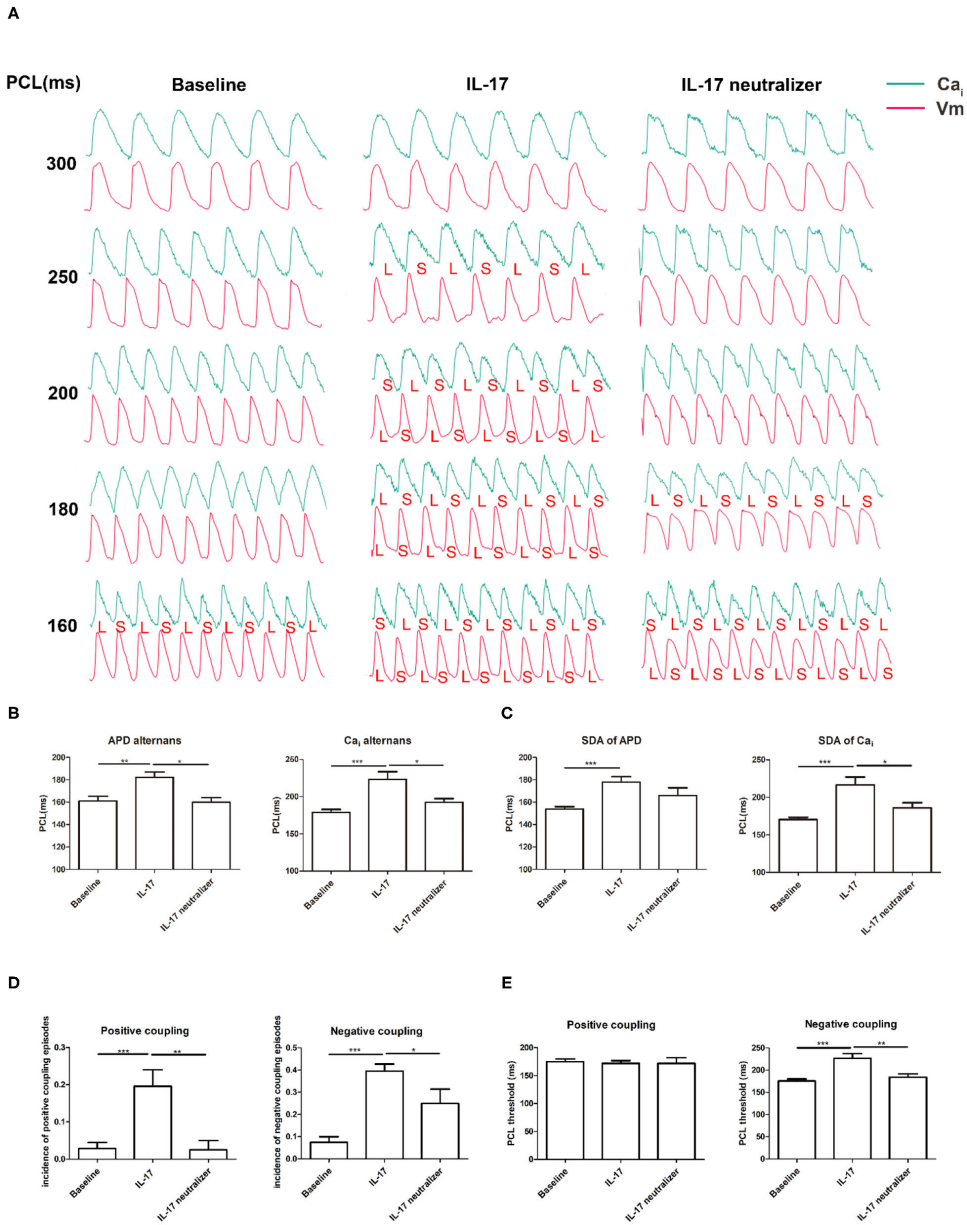
Effects of PCL on APD and Ca_i_TD alternans. **(A)** The green and red tracings indicated the Ca_i_ and Vm signals, respectively. L, long; S, short. **(B)** APD and Ca_i_ alternans. **(C)** SDA of APD and Ca_i_. **(D)** Incidence of positive and negative coupling of Ca_i_-Vm alternans. **(E)** PCL threshold of positive and negative coupling of Ca_i_-Vm alternans. ^*^P < 0.05; ^**^P < 0.01; ^***^P < 0.001. Vm, voltage-driven membrane; SDA, spatially discordant alternans, and other abbreviations as in Figures 1, 2.

An example of increased alternans of APD and Ca_i_TD with decreasing PCL is shown in Figure 3A. In the IL-17 group, Ca_i_ alternans were induced at 250 ms PCL, and APD alternans were detected at 200 ms PCL. In the baseline group, Ca_i_ alternans were induced at 160 ms PCL, whereas APD alternans were not detected. In the IL-17 neutralizer group, significant Ca_i_ alternans were induced at 180 ms PCL, and APD alternans were detected at 160 ms PCL. The PCL threshold of Ca_i_ alternans was greater in the IL-17 group than in the baseline (IL-17 vs. baseline, 223 ± 25 vs. 178 ± 11 ms; *P* < 0.001) and IL-17 neutralizer groups (IL-17 vs. IL-17 neutralizer, 223 ± 25 vs. 192 ± 9 ms; *P* = 0.013). The PCL threshold of APD alternans was greater in the IL-17 group than in the baseline (IL-17 vs. baseline, 182 ± 13 vs. 161 ± 13 ms; *P* = 0.002) and IL-17 neutralizer groups (IL-17 vs. IL-17 neutralizer, 182 ± 13 vs. 160 ± 8 ms; *P* = 0.011) (Figure 3B). No significant difference in the PCL threshold of APD and Ca_i_TD alternans was observed between the baseline and IL-17 neutralizer groups (P = NS). The SDA threshold of Ca_i_ alternans was greater in the IL-17 group than in the baseline (IL-17 vs. baseline, 217 ± 10.5 vs. 171 ± 2.9 ms; *P* < 0.001) and IL-17 neutralizer groups (IL-17 vs. IL-17 neutralizer, 217 ± 10.5 vs. 186 ± 6.7 ms; *P* = 0.003). The SDA threshold of APD alternans was greater in the IL-17 group than in the baseline (IL-17 vs. baseline, 178 ± 4.8 vs. 153 ± 2.1 ms; *P* < 0.001) and IL-17 neutralizer groups (IL-17 vs. IL-17 neutralizer, 178 ± 4.8 vs. 166 ± 6.7 ms; *P* = 0.091) (Figure 3C). IL-17 group had a higher incidence of pacing induced positive coupling of Ca_i_-Vm alternans compared to baseline (IL-17 vs. baseline, 0.20 ± 0.04 vs. 0.03 ± 0.02; *P* < 0.001) and IL-17 neutralizer group(IL-17 vs. IL-17 neutralizer, 0.20 ± 0.04 vs. 0.03 ± 0.03; *P* = 0.004). IL-17 group had a higher incidence of pacing induced negative coupling of Ca_i_-Vm alternans compared to baseline (IL-17 vs. baseline, 0.40 ± 0.03 vs. 0.07 ± 0.03; *P* < 0.001) and IL-17 neutralizer group (IL-17 vs. IL-17 neutralizer, 0.40 ± 0.03 vs. 0.25 ± 0.06; *P* = 0.04) (Figure 3D). No significant difference in PCL threshold of positive coupling of Ca_i_-Vm alternans was be found between the baseline and IL-17 neutralizer groups. The PCL threshold of negative coupling of Ca_i_-Vm alternans was greater in the IL-17 group than in the baseline (IL-17 vs. baseline, 227 ± 10.5 vs. 176 ± 4.4 ms; *P* < 0.001) and IL-17 neutralizer groups (IL-17 vs. IL-17 neutralizer, 227 ± 10.5 vs. 184 ± 7.5 ms; *P* = 0.001) (Figure 3E).

### Effect of IL-17 on the Maximum Slope of APD Restitution Curves and PCL Threshold Triggering Ventricular Tachycardia/Ventricular Fibrillation

**Figure 4:**
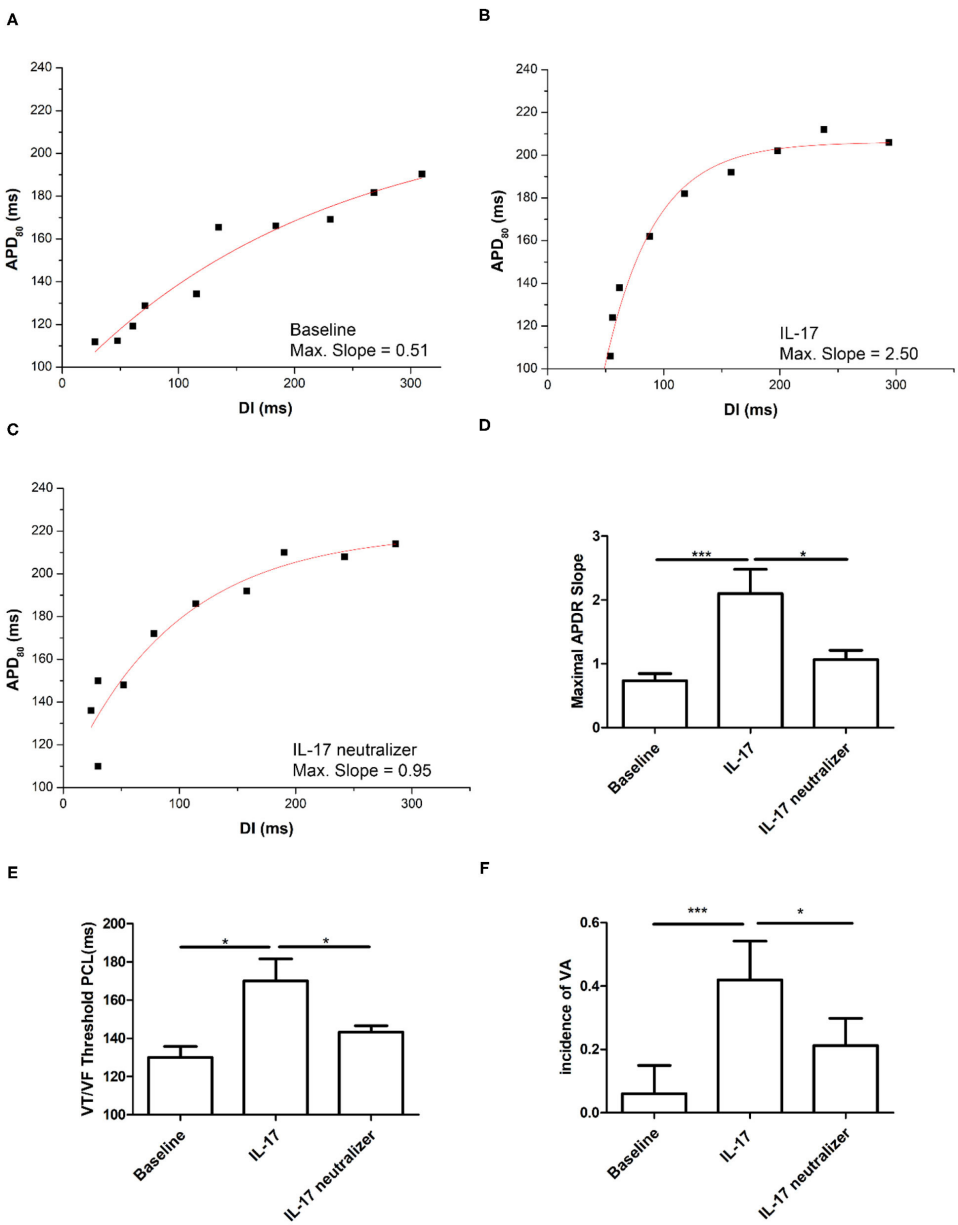
Effects of IL-17 on the maximum slope of APD restitutions and inducibility of ventricular arrhythmia (VA). **(A–D)** Maximum slope of APD restitutions. **(E)** IL-17 on VT/VF threshold PCL. **(F)** Inducibility of VA. ^*^P < 0.05; ^***^P < 0.001. DI, diastolic interval; VA, ventricular arrhythmia; VT/VF, ventricular tachycardia/ventricular fibrillation and other abbreviations as in Figure 2.

The IL-17 group had higher maximum slopes of APD restitution (APDR) curves than the baseline group (2.1 ± 0.8 vs. 0.7 ± 0.4; *P* < 0.001). And, there were significant differences in maximum slopes of APDR curves between the IL-17 and IL-17 neutralizer groups (2.1 ± 0.8 vs. 1.0 ± 0.2; *P* = 0.008) (Figures 4A–D). The PCL threshold triggering ventricular tachycardia/ventricular fibrillation (VT/VF) was higher in the IL-17 group than in the baseline (IL-17 vs. baseline, 170 ± 20 ms vs. 130 ± 10 ms; *P* = 0.01) and IL-17 neutralizer groups (IL-17 vs. IL-17 neutralizer, 170 ± 20 vs. 143 ± 5 ms; *P* = 0.05) (Figure 4E). VA inducibility was higher in IL-17 compared with the baseline and IL-17 neutralizer group (Figure 4F).

### Effect of IL-17 on VF Dynamics in Normal Ventricles

**Figure 5:**
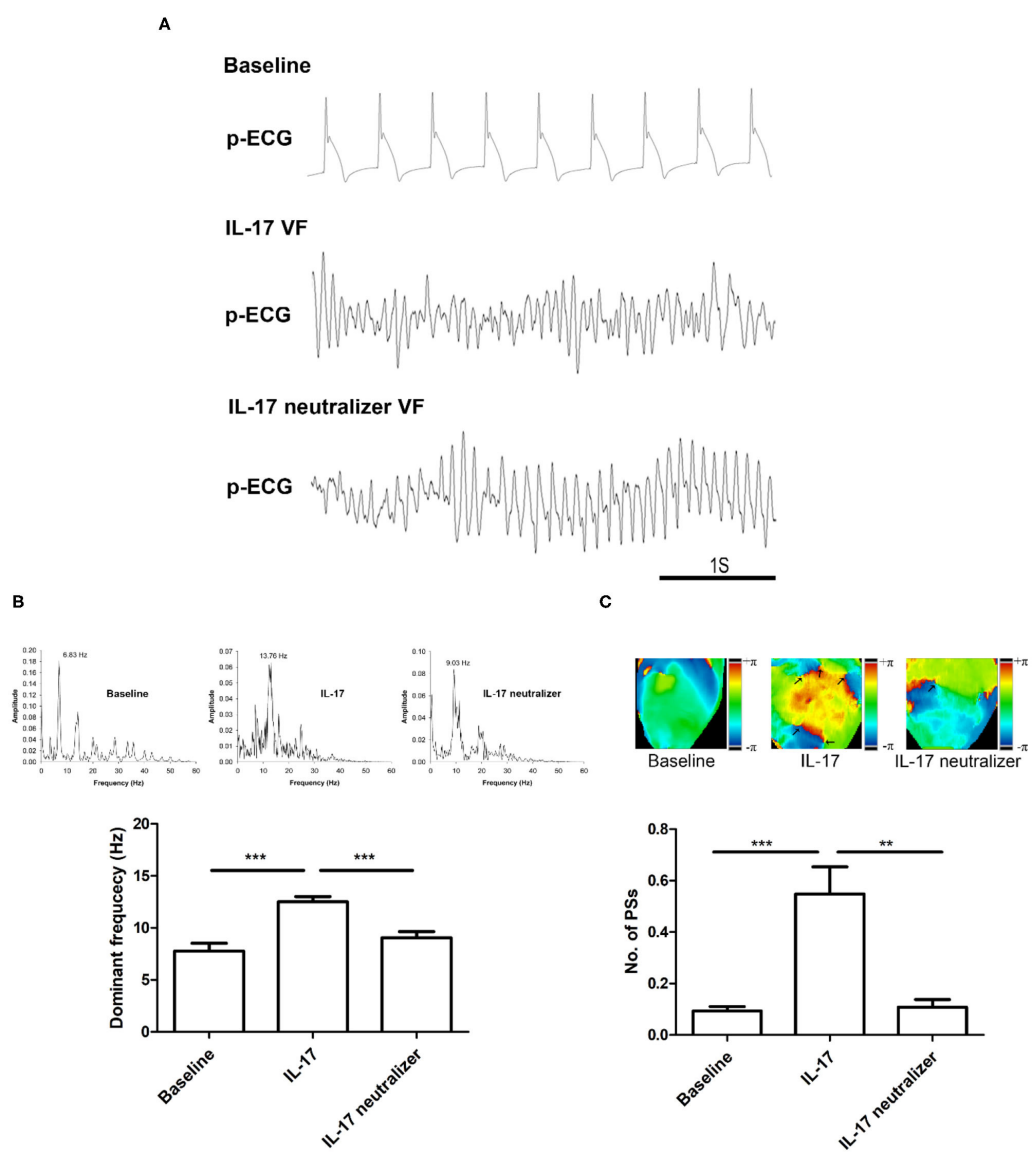
Effects of IL-17 on the DF and wavebreaks of VF in ventricles. **(A)** p-ECG recordings of pacing-induced sustained VF episodes in the baseline, IL-17, and IL-17 neutralizer groups in a normal ventricle. **(B)** Effects of IL-17 on DF of VF in normal ventricles. **(C)** Effects of IL-17 on the number of PSs. ^**^P < 0.01; ^***^P < 0.001. DF, dominant frequency; VF, ventricular fibrillation; PSs, Phase singularities.

Figure 5A shows the p-ECG recordings of VF in the baseline, IL-17 and IL-17 neutralizer groups. The DF of VF was decreased from 13.7 Hz (IL-17 group) to 9.0 Hz after IL-17 neutralizer treatment (IL-17 neutralizer group) compared with the baseline group (6.8 Hz). The IL-17 group had a higher DF of VA than that of the baseline group (IL-17 vs. baseline, 12.5 ± 0.5 vs. 7.7 ± 0.7 Hz; *P* < 0.001) and IL-17 neutralizer group (IL-17 vs. IL-17 neutralizer, 12.5 ± 0.5 vs. 9.0 ± 0.5 Hz; *P* < 0.001) (Figure 5B). Phase maps sampled during VF were analyzed for PSs (wavebreaks). Figure 5C shows phase maps with PSs (black arrows) of the baseline, IL-17 and IL-17 neutralizer groups in LV. The IL-17 group increased PSs than the baseline group (IL-17 vs. baseline, 0.5 ± 0.10 vs. 0.1 ± 0.01; *P* < 0.001) and IL-17 neutralizer group (IL-17 vs. L-17 neutralizer, 0.5 ± 0.10 vs. 0.1 ± 0.02; *P* < 0.001).

### The mRNA Expression of Ion Channels in IL-17-Treated Rabbit Hearts

**Figure 6:**
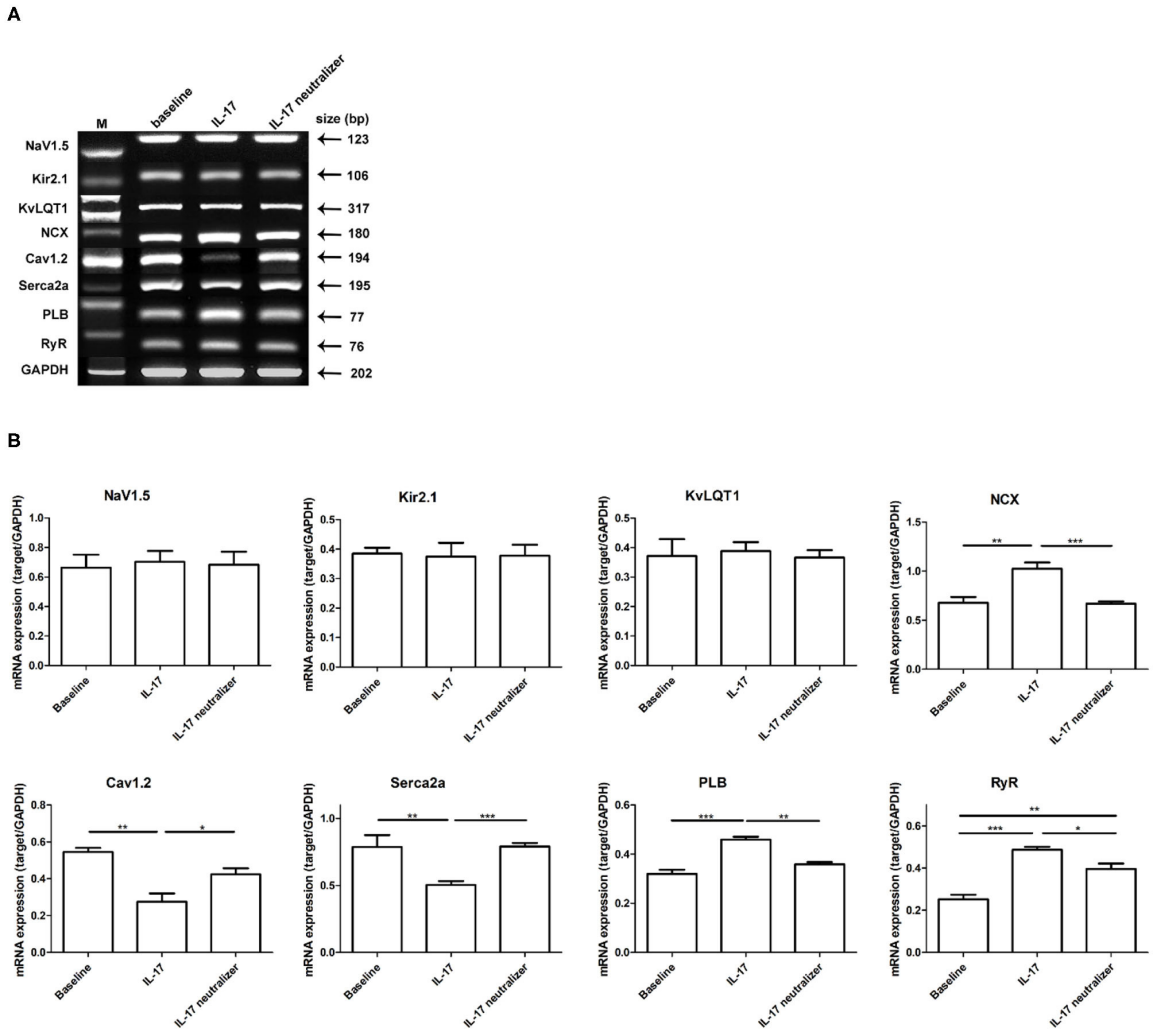
Expressions of various ion channels target in left ventricular. Relative quantities of mRNA levels by semiquantitative RT-PCR were shown among the 3 groups. **(A)** Shows RT-PCR gels for Nav1.5, Kir2.1, KvLQT1, NCX, Cav1.2, SERCA2a, PLB, and RyR. **(B)** Demonstrates the relative mRNA expression quantity. Each value represents the mean ± SD of 3 or more repeat experiments. ^*^P < 0.05; ^**^P < 0.01; ^***^P < 0.001. KvLQT1, subunit of *I*_Ks_; Nav1.5, α1-subunit of Na channel; Cav1.2, subunit of *I*_CaL_; NCX, sodium-calcium exchanger; Kir2.1, subunit of IK1; RyR, ryanodine receptor Ca^2+^ release channel; PLB, phospholamban; SERCA2a, sarcoplasmic reticulum Ca^2+^-ATPase; mRNA, messenger ribonucleic acid; RT-PCR, reverse transcription polymerase chain reaction.

Using semiquantitative reverse transcription polymerase chain reaction (RT-PCR), we found mRNA levels that the Na^+^/Ca^2+^ exchanger (NCX), phospholamban (PLB), and ryanodine receptor Ca^2+^ release channel (RyR) were significantly upregulated in the IL-17 group compared with the baseline and IL-17 neutralizer groups. The mRNA level of RyR was significantly reduced in the baseline group compared with the IL-17 neutralizer group. The subunit of the L-type Ca^2+^ current (*I*_CaL_) Cav1.2 and sarcoplasmic reticulum Ca^2+^-ATPase (SERCA2a) were significantly reduced in the IL-17 group compared with the baseline and IL-17 neutralizer groups. No significant difference in the mRNA levels of NCX, Cav1.2, SERCA2a, and PLB was found between the baseline and IL-17 neutralizer groups. Furthermore, among the 3 groups, no significant difference in the mRNA levels of the following was observed: the α1-subunit of Na channel, Nav1.5; the subunit of the inward rectifier potassium current (*I*_K1_), Kir2.1; the subunit of the slow delayed rectifier current (*I*_Ks_), KvLQT1 (Figures 6A,B).

## Discussion

### IL-17 Modulates Electrophysiology of LV

Increased proinflammatory cytokine levels are associated with HF, hypertension, arrhythmogenic RV cardiomyopathy, and myocardial ischemia (1, 15, 16). In several case-controlled studies, increased levels of inflammatory markers, such as CRP, IL-6, IL-8, and TNF, and elevated neutrophil and lymphocyte ratios have been reported in patients with arrhythmia compared with those in patients with sinus rhythm (17). The inflammation process enhanced by HF is associated with the alteration of ionic currents and the Ca_i_ transient, which predisposes to VA (15). Electrical remodeling prolonged APD and steepened the maximum slope of APDR, which promotes dynamical instability, wavebreaks, and VF (4, 18). Mediators of the inflammatory response can alter electrophysiology and structural substrates, thereby promoting arrhythmia susceptibility. The proinflammatory cytokine such as TNF-α and IL-1β can decrease the SERCA2 expression, which prolongs Ca_i_TD and APD (19). The participation of different inflammation-related cytokines and chemokines has been proposed in the pathophysiology of arrhythmia (4, 19). We previously reported treatment IL-17 directly induced VA in a dose-dependent manner (4). In agreement with previous studies, our present study showed that IL-17 prolonged APD and Ca_i_TD and steepened the maximum slope of APDR, which may result in VA.

### Effects of IL-17 on Electrical Alternans

APD and Ca_i_ alternans are related with arrhythmogenesis, where SDA between myocytes amplify repolarization gradients to produce conduction block and reentrant excitation (20). In our present study, IL-17 enhanced the Ca_i_ and APD alternans and decreased Ca_i_ transient, which may increase VA susceptibility. In the HF model, a decrease in Ca_i_ transient amplitude and contractile dysfunction can be produced by Ca^2+^ leak through the sarcoplasmic reticulum (SR) Ca^2+^ channel RyR and/or reduced activity of SERCA2a (21). TNF-α causes abnormal Ca_i_ handling and arrhythmogenicity in pulmonary vein cardiomyocytes, and it can reduce the mRNA expression of SERCA2a by enhancing methylation in the promoter region (22). IL-1β significantly reduces the contractility of cardiomyocytes and the amplitude and speed of Ca_i_ transients, and it encourage SR Ca^2+^ leak and spontaneous arrhythmic activity when they interact with other inflammatory cytokines (19). Two major mechanisms for the growth of SDA have been proposed: voltage- and Ca^2+^-driven mechanisms (23). The first mechanism was purely Vm potential-driven, which was coupled through the dynamic interaction between the APDR curve and CV restitution curve; however, the Ca^2+^-driven mechanism was considered to be more complex, with discordant alternans produced by instabilities in Ca_i_ cycling that impact APD through Ca_i_-Vm coupling (24). Ca_i_-Vm coupling depended on a dynamic balance between the influx through *I*_CaL_ and extrusion through the NCX current (*I*_NCX_) (23). If the effect of *I*_NCX_ is dominant, positive Ca_i_-Vm coupling occurs, where increased Ca_i_ induces prolonged APD by enhancing Ca^2+^ extrusion through *I*_NCX_. If Ca-dependent inactivation of *I*_CaL_ dominates, a large Ca_i_ transient will rapidly inactivate *I*_CaL_ and tend to shorten APD (24). Electrical alternans have previously been attributed to the disturbances in Ca_i_ signaling, and APD alternans are considered to be a secondary consequence (24).

### IL-17 Modulates Calcium Handling

In our study, mRNA levels of Cav1.2 and SEARCA2a were downregulated in the IL-17 group, and IL-17 neutralizer treatment reversed these changes. The increase in mRNA expression of NCX during HF was found to be associated with imperfect SERCA2a function. Upregulated NCX activity leads to APD prolongation and repolarization instability during HF (25). In the present study, NCX was upregulated in the IL-17 group, and IL-17 neutralizer treatment reversed the change in expression of NCX caused by IL-17. A previous study showed that PLB ablation in TNF1.6 mice (TKO mice) improved contractile function and Ca_i_ transients in isolated cardiomyocytes (26). TNF-α-induced caspase-8 activation results in the leakage of RyR2 channels that promote cardiac remodeling after myocardial ischemia/reperfusion (27). Increased RyR sensitivity and reduced *I*_K1_ contributes to sustained focal arrhythmia in rabbits (28). In agreement with previous reports, our present study demonstrated that the mRNA expressions of PLB and RyR were upregulated in the IL-17 group, and IL-17 neutralizer treatment downregulated these expressions to the baseline, suggesting that abnormal Ca_i_ handling caused by IL-17 can result in VA. In our previous study, the expression of IL-17 via activating MAPK pathway might play an important role in generating VA in ischemic HF. Moreover, our present study showed that IL-17 enhanced Ca_i_TD and APD alternans through the disturbances in calcium handling, which may increase VA susceptibility in normal substrate.

### Possible Mechanism and Clinical Implication

**Figure 7:**
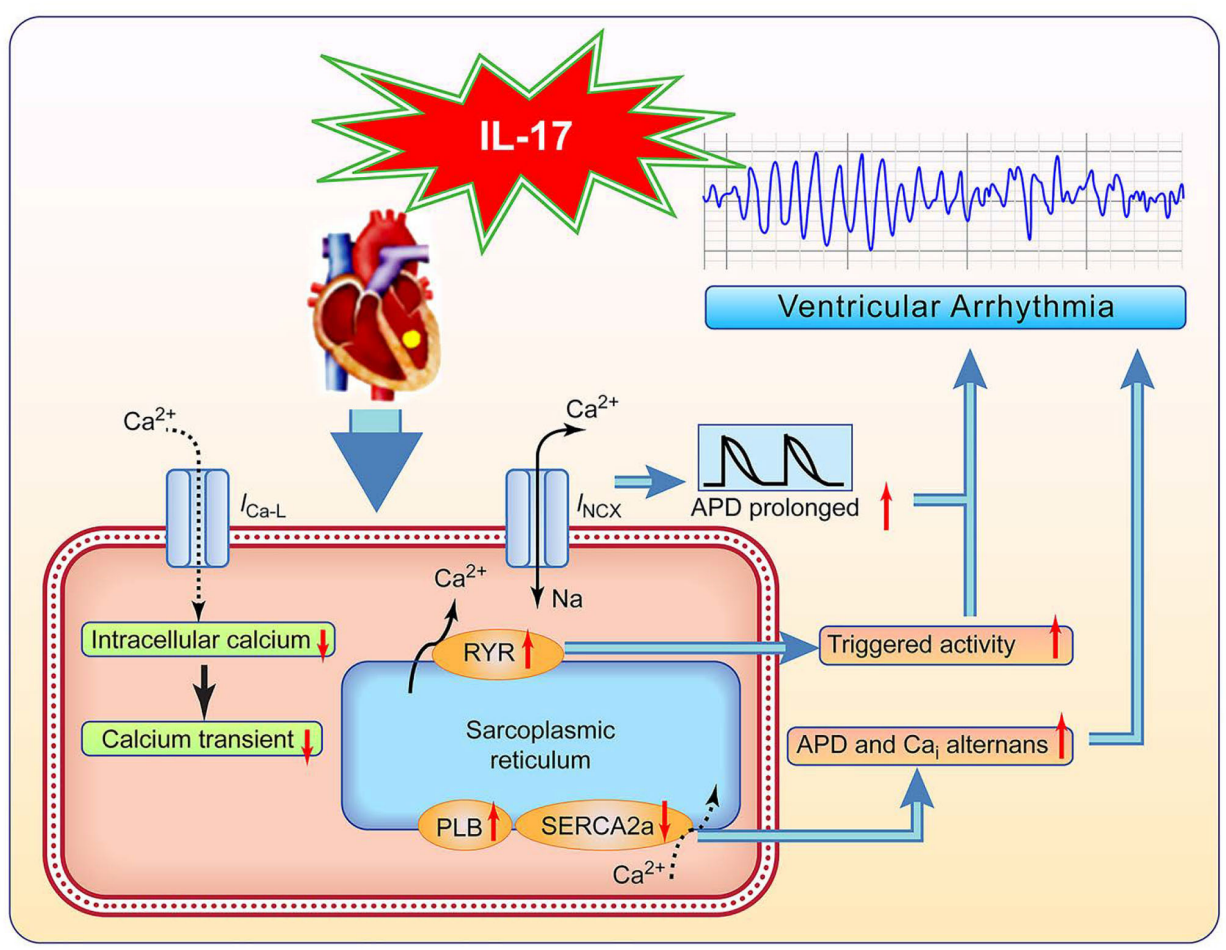
Proposed pathway of IL-17 effects on ventricular arrhythmia (VA). *I*_CaL_, L-type calcium current; *I*_NCX_, Na^+^-Ca^2+^ exchanger current; and other abbreviations as in Figures 2, 3, 6.

We propose a possible mechanism for IL-17–induced VA (Figure 7). IL-17 administration decreased the mRNA expression of Cav1.2 and enhanced that of PLB, resulting in decreased Ca_i_ and Ca_i_ transient. IL-17 administration increased NCX activity contributing to APD prolongation, repolarization instability, and increased RyR sensitivity. Decreased SERCA2a levels may result in susceptibility to APD and Ca_i_ alternans with IL-17 administration. Therefore, IL-17 causes electrical and structural remodeling, resulting in VA. In ischemic HF patients, IL-17 levels may function as a biomarker for monitoring the incidence of VA. A recent study demonstrated that anti-inflammatory therapy targeting IL-1β suppression decreased cardiovascular events in myocardial ischemia patients (16). IL-17 suppression may provide a new therapy to prevent VA in ischemic HF patients.

### Limitation

First, small sample size may cause an insignificant result of IL 17-neutralizer on calcium transients compared to those on baseline. With more statistical power a significant result might be obtained. It is also possible that IL-17 might cause a reduction in Ca_i_ transients through multiple mechanisms rather than the IL-17 receptor alone. Both IL-17 and IL-17 neutralizer can not be washed out. There was no time control in this study. We only examined mRNA expression of ionic channels and calcium handling. It is difficult to directly link the gene expression and functional data. The RNA transcriptions of calcium handling proteins may not fully explain the results observed during measurements of calcium transients. Further study is needed to clarify this issue. Cytochalasin-D has been reported to affect repolarization/conduction patterns and Ca_i_. These effects might interfere with the current results in our rabbit model. A 2 ms frame rate might not be optimal for determining activation/rise time characteristics, and a higher frame rate is optimal.

## Conclusions

Enhanced electrical alternans and abnormal Ca_i_ handling caused by IL-17 can increase susceptibility of VA in normal rabbit heart. Suppression of IL-17 may reverse the adverse effect, providing a potential treatment for VA.

## Data Availability Statement

The original contributions presented in the study are included in the article/Supplementary Material, further inquiries can be directed to the corresponding author/s.

## Ethics Statement

The animal study was reviewed and approved by Institutional Animal Care and Use Committee of Taipei Veterans General Hospital.

## Author Contributions

Y-NT: conceptualization, investigation, methodology, and writing—original draft. Y-WH: conceptualization, validation, and data curation. S-FL: software and resources. Y-HC and Y-CH: formal analysis. W-HT, H-YL, and T-JW: resources. A-SL: methodology. Y-TH: data curation. T-FC and SH: funding acquisition. S-LC: resources, funding acquisition, project administration, and supervision. S-AC: writing—review and editing, supervision, and funding acquisition. All authors contributed to the article and approved the submitted version.

## Conflict of Interest

The authors declare that the research was conducted in the absence of any commercial or financial relationships that could be construed as a potential conflict of interest.
