# Supplementary material for: Proinflammatory Cytokine Modulates Intracellular Calcium Handling and Enhances Ventricular Arrhythmia Susceptibility
Source: Front Cardiovasc Med. 2021 Mar 16;8:623510. doi: 10.3389/fcvm.2021.623510 (PMC8007768; doi:10.3389/fcvm.2021.623510)
Supplement: Supplementary file 1 [file Table_1.DOCX]

Supplementary Material

# Supplementary Table 1: Sequence of forward and reverse primers for RT-PCR

|  |  | **NCBI** |
| --- | --- | --- |
| **Gene** | **Primer (5′**→ **3′)** | **Accession No.** |
| KvLQT1 | F: GCCGCAGCAAGTATGTCG | XM_008252197 |
|  | R: CCTTCTCAGCAGGTACACGA. |  |
| Nav1.5 | F: CTGGAACATCTTCGACAGCA | XM_017340134 |
|  | R: GACTTGGCCAGCTTGAAGAC |  |
| Cav1.2 | F: ATGAAGGCATGGATGAGGAG | NM_001136522 |
|  | R: GCACTTTCTCCTGCAGAACC |  |
| NCX | F: GATGAGAGGGACCAGGATGA | NM_001170958 |
|  | R: CATCAGGCGTGTAGCTTGAA |  |
| SERCA2a | F: CAGCAGATTAATTGGCAGCA | NM_001089321 |
|  | R: GGGTGCAAACCAAGAACACT |  |
| Kir2.1 | F: CAGACACTCCCCCTGACATT | NM_001082198 |
|  | R: CCAGAGAAGGAGTCGGTCAG |  |
| PLB | F: CTCAACAAGCACGTCAAAACCT | NM_001082621 |
|  | R: GCAGATCAGCAGGAGACATATCA |  |
| RyR | F: CACCAGCTATGAGAGGTTCAACA | NM_001082757 |
|  | R: GCTAGGGCCAACTCATTATTATCC |  |
| GAPDH | F: AGGTCATCCACGACCACTTC | NM_001082253 |
|  | R: GTGAGTTTCCCGTTCAGCTC |  |

KvLQT1, subunit of slow delayed rectifier current [*I*_Ks_]; Nav1.5, α1-subunit of Na channel; Cav1.2, subunit of L-type calcium current [*I*_CaL_]; NCX, Na^+^/Ca^2+^ exchanger; SERCA2a, sarcoplasmic reticulum Ca^2+^-ATPase; Kir2.1, subunit of inward rectifier potassium current [*I*_K1_]; PLB, phospholamban; RyR, ryanodine receptor Ca^2+^ release channel; GAPDH, Glyceraldehyde 3-phosphate dehydrogenase.
